# Supplementary material for: Genome-Wide Identification and Expression Pattern Analysis of the WNK Gene Family in Apple under Abiotic Stress and Colletotrichum siamense Infection
Source: Int J Mol Sci. 2024 Aug 5;25(15):8528. doi: 10.3390/ijms25158528 (PMC11313067; doi:10.3390/ijms25158528)
Supplement: Supplementary file 1 [file ijms-25-08528-s001.zip › Supplementary Figure S1.pdf]

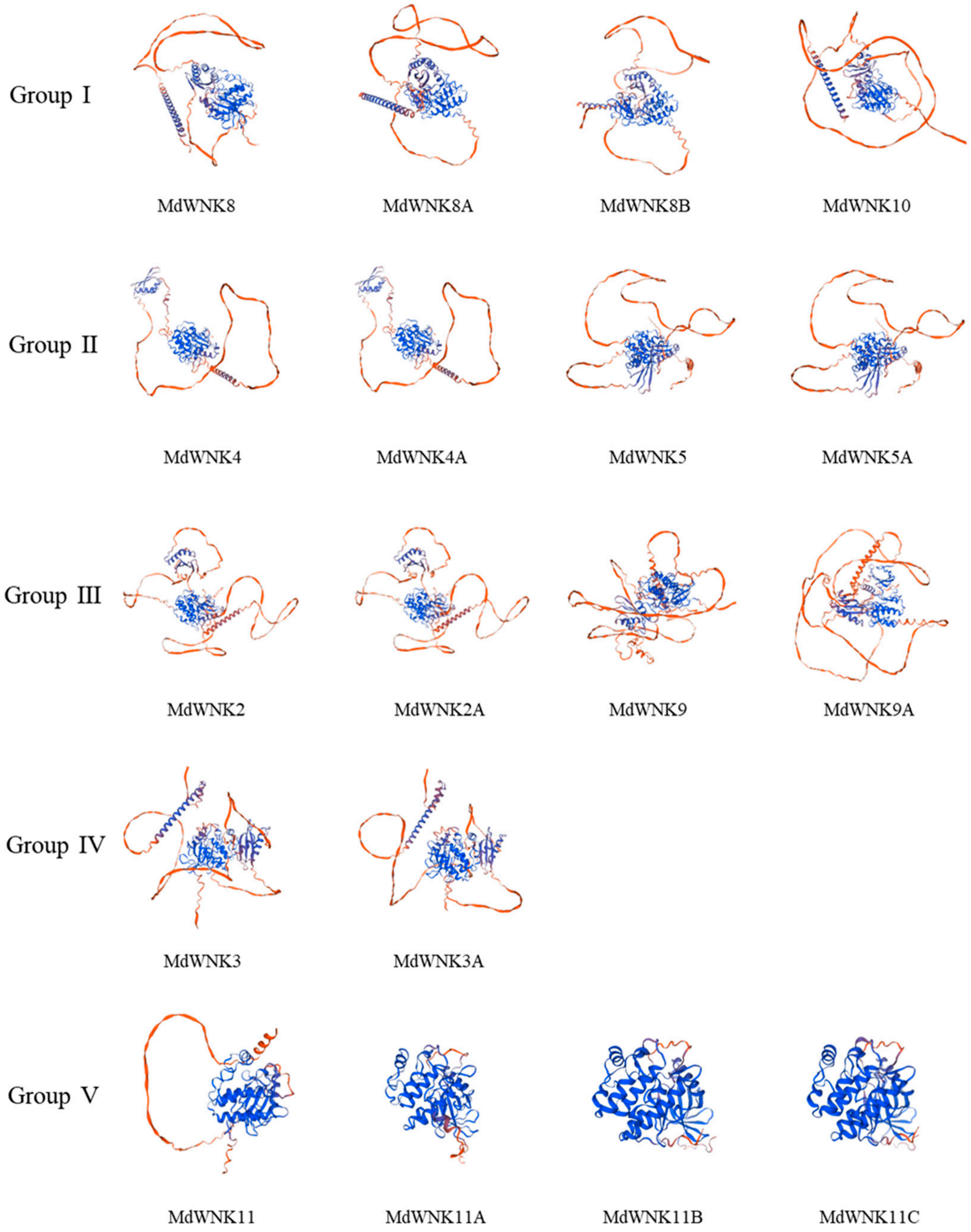

Supplementary Figure S1. Homologous modeling prediction of the 3D structures of the MdWNK proteins. These proteins were divided into five groups by phylogenetic relationships.
