# Supplementary material for: Genome-Wide Identification and Expression Pattern Analysis of the WNK Gene Family in Apple under Abiotic Stress and Colletotrichum siamense Infection
Source: Int J Mol Sci. 2024 Aug 5;25(15):8528. doi: 10.3390/ijms25158528 (PMC11313067; doi:10.3390/ijms25158528)
Supplement: Supplementary file 1 [file ijms-25-08528-s001.zip › Supplementary Figure S2.pdf]

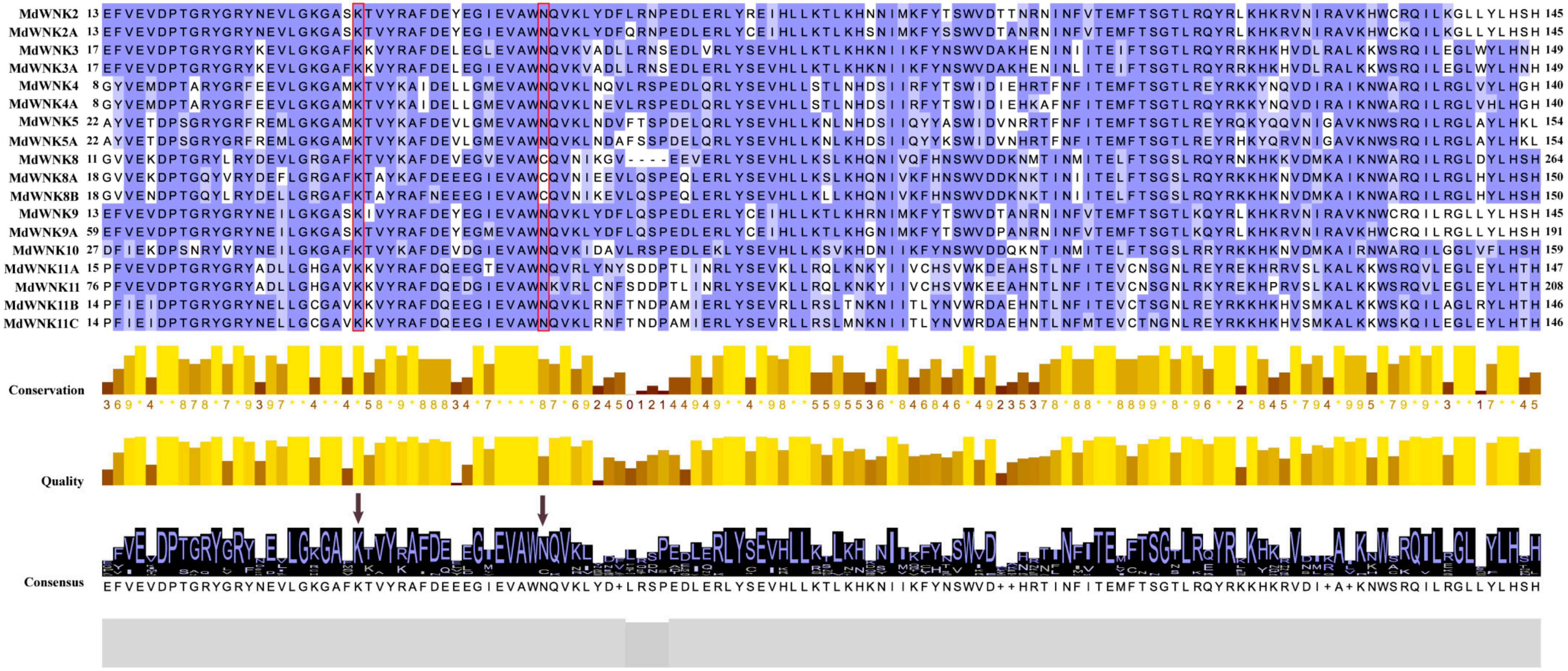

Supplementary Figure S2. Multiple sequence aligment for amino acids in the Protein kinase domains of all MdWNK proteins. The red boxes and gray arrows point to the location of lysine (K) in subdomain I and the location of substituted lysine (K) in subdomain II.
