# Supplementary material for: Genome-Wide Identification and Expression Pattern Analysis of the WNK Gene Family in Apple under Abiotic Stress and Colletotrichum siamense Infection
Source: Int J Mol Sci. 2024 Aug 5;25(15):8528. doi: 10.3390/ijms25158528 (PMC11313067; doi:10.3390/ijms25158528)
Supplement: Supplementary file 1 [file ijms-25-08528-s001.zip › Supplementary Table S1.pdf]

Supplementary Table S1 The secondary structure of WNK proteins in apple

Protein	α-helix	β-turn	Random coil	Extended strand
MdWNK2	35.55%	4.61%	49.12%	10.72%
MdWNK2A	33.33%	4.61%	51.63%	10.43%
MdWNK3	36.39%	3.96%	50.32%	9.34%
MdWNK3A	37.66%	3.96%	49.05%	9.34%
MdWNK4	37.24%	3.40%	50.00%	9.35%
MdWNK4A	38.12%	4.27%	46.67%	10.94%
MdWNK5	36.75%	3.81%	49.67%	9.77%
MdWNK5A	37.48%	3.65%	48.09%	10.78%
MdWNK8	40.10%	5.38%	41.56%	12.96%
MdWNK8A	42.24%	4.13%	43.73%	9.90%
MdWNK8B	39.67%	4.13%	47.44%	8.76%
MdWNK9	34.36%	3.62%	51.95%	10.07%
MdWNK9A	40.64%	4.55%	44.01%	10.79%
MdWNK10	34.81%	3.51%	48.70%	12.98%
MdWNK11	42.57%	6.29%	37.71%	13.43%
MdWNK11A	48.79%	7.61%	31.83%	11.76%
MdWNK11B	49.49%	7.80%	32.54%	10.17%
MdWNK11C	46.78%	7.12%	33.56%	12.54%
