# Supplementary material for: Genome-Wide Identification and Expression Pattern Analysis of the WNK Gene Family in Apple under Abiotic Stress and Colletotrichum siamense Infection
Source: Int J Mol Sci. 2024 Aug 5;25(15):8528. doi: 10.3390/ijms25158528 (PMC11313067; doi:10.3390/ijms25158528)
Supplement: Supplementary file 1 [file ijms-25-08528-s001.zip › Supplementary Table S2.pdf]

Supplementary Table S2 Segmentally duplicated apple *WNK* gene pairs and their Ka/Ks ratios

Seq_1 ID	Seq_1 Name	Seq_2 ID	Seq_2 Name	Ka	Ks	Ka/Ks	Note
MD03G1189000	WNK11B	MD09G1121400	WNK11A	0.1823715	NaN	NaN	High Sequence Divergence Value (pS>=0.75)
MD09G1121400	WNK11A	MD11G1205200	WNK11C	0.1706354	NaN	NaN	High Sequence Divergence Value (pS>=0.75)
MD09G1121400	WNK11A	MD17G1112400	WNK11	0.0274386	0.1375585	0.1994685	
MD03G1053500	WNK4A	MD04G1172700	WNK5A	0.2589545	NaN	NaN	High Sequence Divergence Value (pS>=0.75)
MD03G1053500	WNK4A	MD11G1055100	WNK4	0.0353915	0.1791062	0.1976005	
MD03G1053500	WNK4A	MD12G1186000	WNK5	0.2657651	6.6133066	0.0401864	
MD04G1220500	WNK9	MD12G1236900	WNK9A	0.0532528	0.1427033	0.3731711	
MD01G1085200	WNK10	MD06G1114800	WNK8B	0.311512	1.6708674	0.1864373	
MD01G1085200	WNK10	MD14G1136200	WNK8A	0.3236574	1.715058	0.1887151	
MD03G1189000	WNK11B	MD17G1112400	WNK11	0.1853419	3.1454503	0.0589238	
MD11G1205200	WNK11C	MD17G1112400	WNK11	0.1736025	2.6353793	0.0658738	
MD04G1172700	WNK5A	MD11G1055100	WNK4	0.2700395	NaN	NaN	High Sequence Divergence Value (pS>=0.75)
MD11G1055100	WNK4	MD12G1186000	WNK5	0.2486764	NaN	NaN	High Sequence Divergence Value (pS>=0.75)
MD03G1189000	WNK11B	MD11G1205200	WNK11C	0.0250207	0.2337876	0.1070233	
MD06G1114800	WNK8B	MD14G1136200	WNK8A	0.0568871	0.1718989	0.3309337	
MD06G1165300	WNK3A	MD14G1170800	WNK3	0.0370813	0.1353117	0.2740434	
MD13G1192500	WNK2	MD16G1193000	WNK2A	0.0399411	0.1885945	0.2117827	
MD04G1172700	WNK5A	MD12G1186000	WNK5	0.0461262	0.1394598	0.3307489	
