# Supplementary material for: Genome-Wide Identification and Expression Pattern Analysis of the WNK Gene Family in Apple under Abiotic Stress and Colletotrichum siamense Infection
Source: Int J Mol Sci. 2024 Aug 5;25(15):8528. doi: 10.3390/ijms25158528 (PMC11313067; doi:10.3390/ijms25158528)
Supplement: Supplementary file 1 [file ijms-25-08528-s001.zip › Supplementary Table S3.pdf]

Supplementary Table S3 Primer used for the relative expression of *WNK* genes in apple

Gene	Forward primer (5′-3′)	Reverse primer (5′-3′)
MdWNK2	TGCGGCGTTTTGTTGAGAAG	CATCGTCTTCTCTCCGCCTC
MdWNK2A	TGAGGTGCAGCGTTTTGTTG	CATCGTCTTCTCTCCGCCTC
MdWNK3	CCGCAAGTACTGGTGTGACT	TGCGGATCAGTAGCTGCATT
MdWNK3A	TGGCGGATCTACTACGCAAC	CTGAACGAGCCTGTCGAAGA
MdWNK4	ATGGACCTCACCGTCCTTCT	ATGTTTGTCTCCTCCTGCGG
MdWNK4A	TCAGGTAGACATCCGAGCCA	TTGTGCAGGGTTGACACACT
MdWNK5	GCCTTTGGCATGTGTGTGTT	GCCTCCACGACTGGATCATT
MdWNK5A	ACACGCCCACAGTGTCATAG	CTTGTGTGCCGGTAACCTCT
MdWNK8	AGTCGAAGTAGCTTGGTGCC	TGACAATCGCCAAACCGAGA
MdWNK8A	GCATGGTGTTCCTACTGCCT	TCCCCCTTTAGCTCACCGTA
MdWNK8B	CTAGTACCCGGCTGGAAACC	TACTCTTCTCCCTGACCGCA
MdWNK9	CAAGCTGACGAGTCTGAGCA	GCTCATCTCGAAGCTCCCTC
MdWNK9A	ATGAGTGGGGGTATCAGCCT	GACTGCCAATCGGGTACCAA
MdWNK10	CTCGCAGTGTGATTGGGACT	GAGGCCCGGACTTTGGTAAA
MdWNK11	GCCTGGAATAAGGTCCGGTT	CACTTGGCCAGTATTCCCGT
MdWNK11A	GGACGAGGCACATAGCACTT	GGGTGCCATATACTCGGGTG
MdWNK11B	CTACTCCGAGGTTCGGCTTC	GCAGCCAATCCCAAATCACC
MdWNK11C	CTGAGGTTCGGCTTCTCAGG	TCTGGAGTCCCTAACACCGA
MdActin	ATTCAAGTATGCCTGGGTGC	CAGTCAGCCTGTGATGTTCC
